# (Dis)Obedience in U.S. American Young Adults: A New Way to Describe Authority Relationships

**DOI:** 10.5964/ejop.v14i2.1314

**Published:** 2018-06-19

**Authors:** Maura Pozzi, Alessandro Quartiroli, Sara Alfieri, Francesco Fattori, Carlo Pistoni

**Affiliations:** a Department of Psychology, Università Cattolica del Sacro Cuore, Milan, Italy; b Department of Psychology, University of Wisconsin – La Crosse, La Crosse, WI, USA; c UCD School of Nursing, Midwifery and Health Systems – University College Dublin, Dublin, Ireland; Department of Psychology, Webster University Geneva, Geneva, Switzerland; University of Liverpool, Liverpool, United Kingdom

**Keywords:** obedience, disobedience, social representation, cultural comparison, young adults

## Abstract

The present research aims to investigate the psychosocial phenomena of obedience and disobedience in young adults residing in the United States, as a replication of a previous study by Pozzi, Fattori, Bocchiaro, and Alfieri (2014). We utilize social representation theory as a means to better understand and define (dis)obedience, a behavioral dimension of the concept of authority. The analysis was conducted using a concurrent mixed methods design. One hundred and fifty-one participants completed a self-report online questionnaire. The results indicate that participants see both obedience and disobedience as related to an authority. Obedience was mostly perceived as an ability to be responsive to laws, social norms, or physical authorities, as well as a positive social object. Disobedience, instead, was defined as a failure of a negative line of conduct. These results differ from previous research, contributing meaningfully and pragmatically to the theoretical debate on (dis)obedience. Theoretical and practical implications of these findings are discussed.

Authority relationship, obedience, and disobedience are fundamental components of everyday life within family dynamics (Bray & Harvey, 1992; Darling, Cumsille, & Loreto Martínez, 2007; Fromm et al., 1936), the workplace (Özcan, 2014) and school (Xiao, 1999). As such, social scientists have been investigating these topics for decades (Fromm, Horkheimer, Mayer, & Marcuse, 1936; Moraro, 2014). Within the field of social psychology, Stanley Milgram was the first person to study issues of authority in human relationships, as he examined obedience beginning in 60s (Milgram, 1963). Milgram tried to explain why persons obey (or disobey) an authority’s unjust requests. This study now represents a milestone in the field of social psychology, as over the last 50 years, it has inspired several replications through experimental research design (e.g., Brannigan, Nicholson, & Cherry, 2015; Burger, 2009) and has been connected to diverse issues such as morality (Kohlberg, 1985) and citizenship development (Marzana, Marta, & Pozzi, 2012a, 2012b). All studies examining obedience and disobedience assume Milgram's definition, originating from an everyday use: “If Y follows the command of X we shall say that he has obeyed X; if he fails to carry out the command of X, we shall say that he has disobeyed X” (Milgram, 1965, p. 58).

Traditional literature on (dis)obedience has assumed and verified that Milgram’s assumption and definition of (dis)obedience is valid worldwide (Benjamin & Simpson, 2009; Blass, 2009; Burger, 2009; Miller, 2009; Reicher, Haslam, & Miller, 2014). The choice of a positivist method to study a phenomenon such as (dis)obedience, left a dearth of understanding about authority relationships. Although this initial deductive approach to (dis)obedience has increased our understanding of this topic and played an important role in the current literature (e.g., Passini & Morselli, 2009; Reicher et al., 2014), a more inductive approach to the study of these social processes is necessary (Charmaz, 2008; Mörtl & Gelo, 2015). The continuous use of experimental paradigm (Blass, 2012) left uncovered what Doise (1986) theorized as the ideological level of analysis.

Social representation theory (SRT) shines particularly useful light on issues of (dis)obedience (Moscovici, 1961). Social representations (SRs) are defined as set of beliefs, ideas, attitudes, and behavioral intention that persons, belonging to a specific social group, have referring to a social object (e.g. family, power, money) (Moscovici, 1961). For instance, what people mean by (dis)obedience is co-construed by people in their interpersonal relationship in the same social group (i.e., ethnic, political, religious, cultural, professional, scientific). Thus, SRs form a general way of thinking, feeling, and behaving within *particular* group (Abric, 2003; Fattori, Curly, et al., 2015; Fattori, Pozzi, Marzana, & Mannarini, 2015; Gelo et al., 2016; Moscovici, 2011; Pozzi et al., 2014).

Within the context of Italian culture, Pozzi, Fattori, Bocchiaro, and Alfieri (2014) utilized the framework of social representation theory defined (dis)obedience as a socially constructed phenomena. Pozzi et al. (2014) argued that obedience can be seen as a context dependent behavior, neither positive nor negative *per se*. Obedience and disobedience, in the Italian context are related to the concept of authority (individuals, institutions, and society). While mostly considered an uncritical response to laws, social norms, or physical authorities, disobedience was also defined as an active, conscious line of conduct in which some issues related to freedom and society. This advancement highlights the positive and prosocial element of disobedience.

## Social Representation Theory and Its Importance

The pertinence of SRT (Moscovici, 1961) in studying authority relationship has been widely recognized (e.g., Fattori, Curly, et al., 2015; Galli, 2006; Leman & Duveen, 1999; Marzana et al., 2016; Moscovici, 2011; Pozzi et al., 2014). SRT is a complex paradigm theorizing that all the people belonging to a specific social group share and contribute to the co-construction of common conceptions about a social object (Lo Monaco, Piermattéo, Rateau, & Tavani, 2017; Moscovici, 1961). In the present study, the social-object is the authority relationship and its behavioral expressions of obedience and disobedience. These shared representations stem from interpersonal relations, communication, media influence, and the cultural history of the group, and helps people comprehend the world and communicate with others (Moscovici, 1961).

Starting from Moscovici’s theorizations, Abric (1993) developed a structuralist approach, theorizing that a SR is composed by a *content*, a set of beliefs, attitudes, and scripts, and is organized in a precise *structure*. Abric (2003) conceives the representational structure as composed by a central core, called *nucleus*, and a *periphery*. Nuclear elements give unity and stability to the representations, while peripheral elements allow plasticity to the representation, giving persons belonging to the same social group, the possibility to have different conceptions of the same representation. All the information, beliefs, ideas, and attitudes related to a specific object of representation, give meaning to the representation according to their place in the structure. Specifically, nuclear elements are key components defining the social object, while peripheral elements can be behavioral tendencies, the expression of a minority group, or elements fading in or out from the representation (Fasanelli, Galli, & Sommella, 2005). In this perspective, authority relationship can be viewed as a relationship of obedience and disobedience as two social representation objects for two main reasons (Galli, 2006). The first one is that they respect the inclusion criteria defining when a social object can be a social representation object: a) having a strong social relevance; b) being object of social interaction; c) being in relation with other social objects (e.g., via power, dominance, hierarchy); and d) referring to social norms and values strictly connected between them. The second reason is given by the intrinsic and contingent nature of the topic. Obedience and disobedience are phenomena with which people deal every day, often implicitly, throughout their entire lives, and that nowadays are important social issues about which people want to communicate (Chomsky, 2013; Galli, 2006).

At a methodological level, this means that: (1) open-ended self-report questionnaires will be used to collect verbal material about a specific social object - (dis)obedience; (2) the contents depicted in this verbal material should be analyzed with regard of both their frequency of appearance (indicative of how much they are shared within a group) and the rank of relevance (indicative of how much they are important within a group) (Abric, 2003; Fasanelli, Galli, & Sommella, 2005). The result is a matrix organized as follow: (a) A nucleus (high frequency and high rank), representing the *core* and most stable SR features shared by the group. (b) A first periphery (high frequency and low rank), representing less central and more open to change but still representative aspects of the SR. (c) the elements of contrast (low frequency and high rank), which are characteristic of a possible minority within the considered social group; these elements are more open to change than the elements of the first periphery. (d) Finally, a second periphery (low frequency and low rank), representing the least representative characteristics of the minority (a lower amount of individuals) and presents the highest degree of openness and flexibility to change (Abric, 2003; Fasanelli et al., 2005). Some scholars assumed social representations are ruled by opposite concepts of interaction, called *themata* (see Marková, 2003 for a more detailed description). In this research, in accordance with previous results by Pozzi et al. (2014), we choose to adopt a more structural approach such as Abric (2003) suggests.

The need to communicate about obedience and disobedience reaffirm the salience of these two social objects, making them important representational objects. Through interpersonal communication, people share knowledge about authority relationship and obedience and disobedience, shaping beliefs and attitudes useful to take a position and to act properly in the social world. In sum, SR can be seen as tool for communication and to behave in social interactions. It is the transformation of abstract concepts in real guidelines that people can follow to be functional in the social world.

Given these premises, this paper aims to replicate the study by Pozzi et al. (2014) in order to verify if the new definition of (dis)obedience, identified through the SRT, is also applicable in the context where Milgram's original definition was developed. Specifically, we attempt to answer two research questions: a) how can obedience and disobedience as social representation objects be defined within U.S. culture, and b) are there differences between the U.S. and previous results on social representations of (dis)obedience? In this research we hypothesize that US young adults will evoke the same Italian’s SR of (dis)obedience. For Italians obedience is both: (a) the respect for social norms given by an institutional authority and (b) the compliance with orders or requests given by a physical authority. Obedience is constantly assessed (in its value) considering the request and the outcome of the act of obedience. Obedience is often accepted passively and unconditionally by the actor in the authority relationship. Thus, disobedience is intended as a lack of respect of both (a) laws and rules that may be imposed by a physical authority and/or (b) for social norms imposed by social group. Disobedience is always context related, that means that the assessment of a disobedient act depends on the context in which it is implemented and on the outcomes. The disobedience act is always under the subject’s awareness: this means that those who disobey conceive the illegitimacy of a request or the injustice of a rule in a specific situation and decide to oppose to it consciously.

## Method

### Participants

A sample of 151 U.S. young adults (79 female; 52.3%)^i^ aged between 19 and 25 years (*M* = 22.7, *SD* = 1.3) took part in this study on a voluntary basis and signed participation consent forms. Seventy participants (33 female; 47.1%) answered the questionnaire related to obedience (*M* = 22.96; *SD* = 1.481) while 81 participants (46 female; 56.8%) answered the questionnaire related to disobedience (*M* = 22.46; *SD* = 1.078). One hundred and eleven participants (73.5%) identified as university students, 27 (17.9%) identified as student-workers, and 8 (5.3%) indicated they were workers. Eighty-nine (58.9%) participants stated that they have a college degree, 58 (38.4%) indicated having a high school degree, and three (2%) reported having a PhD. Participants were recruited through a convenience sampling procedure in two North American higher education institutions located on both East and West coast. The study was approved by the institutional review boards of the institutions where data collection took place.

### Research Design

A mixed-method research design was conducted. The central premise of using mixed methods is that an intentional and synergic integration of quantitative and qualitative approaches provides a better understanding of research problems than either approach alone, as it provides more comprehensive evidence for studying a research problem, especially when it is very complex and asks for multiple sources of data (Creswell & Plano Clark, 2011). By using a convergent parallel mixed-method design, the intent of the research is to concurrently collect both quantitative and qualitative data, to analyze both datasets, and to integrate the findings in a coherent enriched manner. This process would allow researcher to validate one set of results with the other (Alfieri, 2017; Aresi & Pedersen, 2016; Creswell, 2013; Gelo, Braakmann, & Benetka, 2008). We obtained a data corpus (the content of the representation), treated qualitatively through a thematic analysis (Braun & Clarke, 2006), and a data set (structure of the representation) analyzed quantitatively using Evoc2000 software (Vergès, 2002).

In order to analyze the *content* of the representation, on each single data corpus (one for obedience and one for disobedience), we first labeled the collected open answers (Level 1-coding), then we identified groups of labels which have similar meaning and clustered them identifying main overarching themes (Level 2-coding), and finally explored and analyzed the relationships among the themes into a concise, coherent narrative which “connects” the emerged themes. At first, the four judges independently completed the analysis on both obedience and disobedience, and then, as a group, they finalized the analysis through a negotiation process.

The *structure* of the representation, has been recreated by inserting the terms, results of the free associations, in Evoc2000 (Vergès, 2002). Before this operation, the terms had been clustered into semantic categories or lemmas. These categories are the result of a matching process between all the words with similar meanings according to the explanation given by the participants (“Why have you chosen X?”). According to the structural approach theorization (Abric, 2003; Vergès, 1992), the Evoc 2000 software generates a matrix composed by four quadrants taking into account both frequency and ranking. As Fasanelli et al. (2005) indicate, “only the intersection of these two [qualitative and quantitative] criteria allows for the identification of the statute of constitutive elements of the social representation being studied” (p. 113).

### Instrument

A self-report online questionnaire was administered using Qualtrics online platform^ii^ to college students in two higher education institutions in the United States. This instrument was composed of two sections:

1. an open-ended question framed to investigate the content of the representation (“In your opinion, what is obedience?” and “In your opinion, what is disobedience?”);
2. an exercise of free associations based on the hierarchized evocation technique (Vergès, 1992), to disclose the structure of the representation. Participants were asked to associate five nouns and five adjectives with the inductor word (“*Obedience*” for half of the sample, “*Disobedience*” for the other half) and to rank them by importance. To disambiguate nouns and adjectives, subjects were asked to briefly explain their choice (Fasanelli et al., 2005).

## Results

### Obedience

#### Thematic Analysis

According to the participants in the study, the term obedience evoked an *act of compliance with orders and rules* (Level 2-coding). A common idea presented by the participants was the idea that obedience is the tendency of “doing what one is told.” For example, “Obedience is when you obey certain task, to comply with rules and laws” (Participant 1); “Obedience is when you listen to what is being told” (Participant 2). Participants used different labels to indicate how the authority asks for obedience: *orders^iii^, norms, rules, laws* and *guidelines* (Level 1-coding). Participants defined obedience as “how well you listen to directions, laws or guidelines and how well you follow them” (Participant 69). They also defined it as “willingly following laws, rules, guidelines, and expectations” (Participant 67), as well as “following the rules set by someone else” (Participant 45). Authority was mainly considered in a *one-to-one relationship* (Level 2-coding) (parents, superiors, and teachers). However, a few participants indicate society, as a whole, as an authority, defining obedience as “being able to follow rules or direct commands given by some[one] of higher authority then you” (Participant 10) and as the tendency to follow “the norms of our society” (Participant 19).

Obedience also evokes the theme of *ability* (Level 2-coding), which is something that individuals learn since childhood. Some participants indicated how obedience is “the ability or willingness to follow something” (Participant 50), while others defined it as “one’s ability to remain faithful and committed to something” (Participant 46). Participants conceive different *ways of being obedient* (Level 2-coding), from agreeing and willingly accepting the order to blindly obey. In this context, participants described it as “following directions and accepting to agree about something” (Participant 19), “being able to listen, obey, and confirm with what you are told, but respectively and with no hesitation” (Participant 25), and “doing things that may go against your personal preference in order to support a greater overall good” (Participant 71). *Loyalty* (Level 2-coding), was defined by participants as faithfulness towards people who depend on your action. For example, a participant reported how “being obedient is remaining faithful to those who you care about and to those who depend on you for fulfilling certain wants or needs” (Participant 37). Similarly, another participant defined obedience as “one’s ability to remain faithful and committed to something” (Participant 46). From participants’ answers arises a *positive* view (Level 2-coding) of obedience. For example, while a participant describes obedience as “always doing the right thing. Even if you're told otherwise” (Participant 31), another describes it as “following an order in a good manner and good attitude” (Participant 43).

#### Structure Analysis

The structure analysis of the SR enables the researcher to organize the results in a 4 quadrants table. The upper left quadrant of the output (see Table 1 and 2 respectively for Obedience and Disobedience), characterized by a high frequency and high average rank of appearance, includes the terms *belonging* to the nucleus. These elements are fundamental and stable components that form the central core of the representation, and represent the stability and unity of the SR. The first periphery, located in the upper right quadrant, is characterized by a high frequency of appearance and by a low average rank of appearance: these terms indicate the behavioral tendencies related to the SR (Palmonari & Emiliani, 2009).

**Table 1:** Social Representation Structure – Obedience

Nucleus			First periphery		
Nouns	*f* ≥ 13	Rank < 3	Nouns	*f* ≥ 13	Rank ≥ 3
Family	21	1.7	School	33	3.4
Rules	16	2.2	Animals	25	3.9
Respect	16	2.8	Army	16	3.1
Children	14	2.7			
Religion	13	2.7			
Adjectives	*f* ≥ 13	Rank < 3	Adjectives	*f* ≥ 13	Rank ≥ 3
Positive	30	2.9	Submissive	19	3.5
Respectful	21	2.4			
Compliant	21	2.8			
Faithful	16	2.9			
Obeying	13	1.8			
Loyal	13	2.1			
Elements of contrast			Second periphery		
Nouns	7 ≤ *f* < 12	Rank < 3	Nouns	7 ≤ *f* < 12	Rank ≥ 3
Follower	10	2.2	Superior	10	3.3
Loyalty	10	2.5	Compliance	8	3.0
Work	10	2.7	Law	8	3.2
Police	8	2.0	Conformity	7	3.9
			Attention	6	3.8
			Social-order	6	3.0
Adjectives	6 ≤ *f* < 12	Rank < 3	Adjectives	6 ≤ *f* < 12	Rank ≥ 3
Willing	11	2.1	Strict	10	3.4
Smart	6	2.7	Hard	7	3.3
			Dutiful	6	3.0
			Listened	6	3.7

*Note.* *f* = Frequency.

The elements of contrast located in the third quadrant, on the lower left, are characterized by a low frequency of appearance and by a high average rank of appearance. The terms in this section can characterize the nucleus or symbolize the tendencies of a minority. The second periphery, the lower right quadrant, is characterized by a low frequency of appearance and low average rank of appearance. Here, we found the elements going in or out from the representation. See Table 1 for the structures’ summary and comparison.

### Disobedience

#### Thematic Analysis

For the majority of the participants, disobedience was perceived as the refusal *to obey a law* or an *authority order* (Level 2-coding). Moreover, it is an act against *different types of authority* (Level 2-coding) identified in parents or superiors. For example, one participant reported that disobedience is an act of being “disobedient to your parents, the police, to teachers, or even to God”^iv^ (Participant 12). Participants identified *different types of “orders”* (Level 2-coding) with *commands*, *rules,* and *laws* (Level 1-coding) being the most cited. One participant reported how disobedience “is the lack of obedience or not obeying a rule, law, or an order” (Participant 52). Disobedience evokes the theme of *failure* (Level 2-coding), clearly observed in the participants’ tendency to define disobedience as “failing to obey certain laws to be followed” (Participant 46), “failure to abide by the rules, order, regulation” (Participant 6), and “failure to obey an authoritative figure” (Participant 73). Furthermore, participants reported that disobedience evokes an *intentional* behavior (Level 2-coding), as captured by some of the participants’ statements. For example participants defined disobedience as “intentionally going against what you are expected to do” (Participant 48), “blatant disrespect for the person who you are intentionally undermining” (Participant 62), and “voluntarily and purposefully violating a superiors orders” (Participant 76). Overall, disobedience was perceived as a negative behavior: “defined as misbehavior” (Participant 80), “doing the wrong things” (Participant 87), and “not doing the right thing or being negligent” (Participant 102).

#### Structure Analysis

In analyzing the structure of the social representation of disobedience, see section "Obedience: Structure Analyses" above for the meaning of the quadrants.

**Table 2:** Social Representation Structure – Disobedience

Nucleus			First periphery		
Nouns	*f* ≥ 12	Rank < 3	Nouns	*f* ≥ 12	Rank ≥ 3
Prison	24	2.8	Children	29	3.4
Disrespect	21	2.6	Animals	21	3.3
Criminal	18	2.8	Negativity	19	3.4
			Rebellion	19	3.3
Adjectives	*f* ≥ 10	*Rank* < 3	Adjectives	*f* ≥ 10	*Rank* ≥ 3
Negative	64	2.9	Rebellious	38	3.0
Rude	21	2.8	Naughty	14	3.1
Disrespectful	19	2.2			
Stubborn	16	2.9			
Elements of contrast			Second periphery		
Nouns	7 ≤ *f* ≤ 11	*Rank* < 3	Nouns	7 ≤ *f* ≤ 11	*Rank* ≥ 3
Ignorance	11	2.6	Rule	8	3.1
School	10	2.8	Insubordination	7	3.0
Non compliance	9	2.4	Time-out	7	3.6
Defiant	9	2.6	Troublemakers	6	4.0
Teenager	8	2.7			
Punishment	7	2.3			
Law	7	2.6			
Adjectives	6 ≤ *f* ≤ 9	*Rank* < 3	Adjectives	6 ≤ *f* ≤ 9	*Rank* ≥ 3
Ignorant	9	2.8	Non compliant	9	3.8
Defiant	8	2.1			
Sad	6	2.3			

*Note.* *f* = Frequency.

## Discussion

This section deals with the aggregation and the comparison between thematic and structural analysis of both obedience and disobedience. As previously stated by some structuralist scholars (Abric, 2003; Fasanelli et al., 2005), only from the combination of the content and its structure is it possible to gain the complete meaning of a social representation.

### Obedience – Comparison Content-Structure

We observed a striking similarity between nuclear elements and narrative themes, giving strength and unity to the representation of obedience. The terms *rules* and *law* confirm the necessity of an explicit form of order to evoke obedience. Social norms and laws (e.g., National Constitution, Street Code) have a fundamental role in regulating human behavior in a variety of social contexts (Kelman & Hamilton, 1989; Passini & Morselli, 2009).

Participants considered obedience as resulting from two processes of (non)consciousness: a) a blind answer to a request, as destructive obedience (Blass, 2012; Milgram, 1963), or b) as a conscious agreement to an order. This second process of obedience stems from the correspondence between individual and authority values (Kelman & Hamilton, 1989). For example, if a person shares the values of the authority, then he or she agrees with orders and norms they consider as coherent with the society’s fundamental values (Haslam, Reicher, & Birney, 2014; Reicher, Haslam, & Miller, 2014). Parents, in fact, are fundamental models for children’s attitudes and behaviors, and their influence remains even in adulthood (Alfieri et al., 2014a, 2014b, 2016; Barni et al., 2013; Iafrate, Donato, & Bertoni, 2013).

Furthermore, obedience is also considered as an *ability*, a positive skill a child should learn and act out during his or her lifespan. In fact, participants focused on obedience as the act of obeying figures such as parents and teachers, whom they follow in order to learn how to behave. Terms as *family* and *religion* reinforce this last statement and represent places where a *child*, as evoked in the nuclear elements of the representation, co-construct the SR of obedience. Since Piaget’s (1932) first reflections on the authority relationship, personified by the child-parent interaction, it has been hypothesized that the child experiences autonomy and heteronomy is through the first forms of social relations (i.e., family relations). The child is able to do so, by assessing these forms of thinking in a positive or negative way according to their parents’ values (Leman & Duveen, 1999). When childhood ends and the individual experiences different relational contexts, such as *school*, *the military*, and *work*, he or she learns obedience through socialization processes which are influenced by his or her cultural belonging (Xiao, 1999). This thesis affirms once again with SRT (Moscovici, 1961) and with the importance of studying such phenomena through a societal approach.

Obedience also evokes the idea of respect (*loyal*, *faithful*, *respectful*), as the individual has to respect and be loyal to his or her commitments toward other people. According to this dimension, it is relevant to refer to Kelman and Hamilton’s (1989) study focused on military obedience. In a war context, a person is inclined to obey depending on three variables: (a) how the expectation of his or her social role imposes certain behaviors; (b) how to avoid sanctions and stigmatization; (c) how his or her values align with those conveyed by the authority’s request(s). The dimension of personal respect towards other people is evoked—an obedient person is someone who is reliable.

Finally, the representation of obedience, obtained via merging content and structure, is largely considered as positive (e.g., the most commonly used adjective in the nucleus was *positive*).

### Disobedience – Comparison Content-Structure

A strong correspondence between content and structure emerged from the data, giving us a coherent representation of disobedience as a social object. The term disobedience evokes a lack of respect for the law or for a person’s order. It indicates a refusal to comply with an explicit command, whether in the form of a formal law or a verbal order.

Participants refer to commands in different ways along a continuum, going from very formal laws to verbal, more informal orders. Disobedience evokes the concept of *failure*: a disobedient person is someone who is not able to do what he or she has been told to do. This topic recalls the educative level of obedience, that is, a person who does not learn from his or her family to behave properly in an authority relationship, is going experience more difficulty obeying. A disobedient person is considered uneducated (*ignorance*, *stubborn*, *rude*), because he or she does not respect other people and does not know how to behave in occasions where obedience is required, showing a lack of respect for social norms. Once again, we have concrete subjects of disobedience: *children*, *animals* and *teenagers*, revealing the conception of a one-to-one disobedience.

Another issue in the representation of disobedience is the level of *intentionality*. For the participants in the current study, disobedience is always an intentional behavior. This point seems to contradict the idea of disobedience as failure, because it considers disobedience as a planned and conscious act. This is an issue that accompanied disobedience since its first theorizations (Lefkowitz, 2007; Milgram, 1974). The reflections on conscious disobedience evolved from Milgram’s (1974) conception of disobedience as an escape behavior from a stressing situation. According to Milgram, people who cannot accept the authority’s order feel a cognitive dissonance. Their inability to deal with such dissonance, and the attempt to reduce it, lead them to disobey to the order. However, other scholars began to uncover the processes underlying disobedience, both as historical analysis (Kelman & Hamilton, 1989; Modigliani & Rochat, 1995) and as experimental studies (Bocchiaro & Zimbardo, 2010; Bocchiaro, Zimbardo, & Van Lange, 2012). These studies highlighted how disobedience is a final outcome of a rational process. People challenging the status quo evaluate the authority request or the law as illegitimate, find alternatives to the situations, and finally, if they feel effective, they could disobey. People who cannot accept the authority’s orders experience a cognitive dissonance that leads them to disobey in order to reduce the dissonance. Generally, this disobedience can be perceived as failure.

Participants perceive disobedience negatively, due to its evoking of a sense of wrong doing. Moreover, the negative evaluation of disobedience stands out in the structure, monopolizing all the quadrants with the majority of terms having negative connotations. In this sense, nuclear elements such as *prison* and *criminal* are significant because they highlight the process of a disobedient act, which ends with one of the worst possible consequences and *punishment*: the loss of freedom. From these terms we can infer participants implicitly refer to disobedience as an antisocial behavior, something dangerous for people and for society. Antisocial disobedience has been theorized as a specific type of disobedience (Passini & Morselli, 2013), which favors only one social groups and disadvantages the other groups (e.g., Ku Klux Klan). There is no possibility to highlight a positive side of disobedience, such as pro-social disobedience (Passini & Morselli, 2009).

### Comparison Between Obedience and Disobedience

According to participants’ evocations, obedience and disobedience seem to be two partially overlapping representations, while concurrently being in strong opposition with each other.

To have both obedience and disobedience it is necessary to have “orders” that can assume several forms. It is possible to have explicit order, such as in the contexts of the military or family (as the ones in Milgram’s study in 1963), or implicit orders such as laws (e.g., via the Constitution). Obviously, these representations share the need for real authority figures to give orders or to create laws. Different authorities express themselves through various channels to give orders. For instance, “institutional” authorities can create and establish laws that people must follow, while parents, to fulfill their role, can give verbal indications to their children.

The SR of obedience and disobedience also point out the subjects who can obey or disobey. In both representations, participants refer to *children*, *teenagers,* and *animals* as the main subjects in an authority relationship, indicating a preference in configuring an authority relationship as a face-to-face (one-to-one) relationship. School as a place of obedience and disobedience focuses on children, but also on educative figures such as teachers, who should teach a child how to behave properly and that are to be obeyed. In fact, obedience evokes the concept of ability, something that a person should learn in his or her first stages of life. When a person fails in learning this ability, then disobedience likely stands out. Moreover, while obedience evokes compliance to social norms, there are no references for social or civil disobedience. Another strong difference emerges through the participants’ polarization in the assessment of obedience and disobedience. The former evokes exclusively positive terms and adjectives, while the latter seems to be represented as something negative, for its nature and consequences. These antinomies, represented by the couple *ability-failure* and *positive-negative,* remind us of the dialogical approach within the SRT (Marková, 2003). Namely, assumed social representations are ruled by opposite concepts of interaction, called *themata*. Themata are diads of opposite concepts, such as positive-negative, that serve to interpret the social world. For example, if a child learns that obedience is positive, he or she will learn that everything that is not coherent or similar to obedience is negative. In this way people categorize the social world, creating their own representations deriving from the collective memory of the social group to which they belong.

Regarding our first research question, we offer a definition of obedience and disobedience according to the SRT paradigm (Moscovici, 1961). Obedience is seen as the respect of orders that can be verbal, as parents with children or sergeant with soldiers, or written, as the constitutional laws or the code of the street. These “orders” necessarily come from an authority that can assume different forms, such as a parent, a superior at workplace, or his or her own social group or society in general. Obedience can be blind or critical, depending upon if the obedient person reflects on and agrees with the values underlining the authority request or with the norms imposed by society.

Disobedience, conversely, is a lack of respect of orders, given by an individual authority or of a formalized law, inserted in civil codes. It is defined as a behavior performed by an individual towards another individual representing the authority. Disobedience is always intentional and it is the outcome of a rational, cognitive process. It is defined as a negative behavior, and in its extreme cases can lead to imprisonment.

### Comparison Between U.S. and Previous Results

According to the second aim of the present research, the results highlighted more differences than similarities between previous results (Pozzi et al., 2014 based on an Italian sample) and U.S. participants.

#### Social Representation of Obedience

Italian and U.S. participants commonly evoked and shared three components: 1) *Individual authority*, authority relationship as an interpersonal relationship; 2) *Social learning*, social institutions such as family and school should teach obedience; 3) *Awareness*, a necessity to agree with the authority’s request, in opposition to blind obedience. Undoubtedly, family and school are the first contexts where a person experiences relationships with authorities and its limits (Bray & Harvey, 1992; Fromm et al., 1936; Xiao, 1999). Parents and teachers represent the first authorities and their educative role is fundamental for the functional development of children’s critical thinking and moral reasoning (Kohlberg, 1985; Marzana et al., 2012a). A parental education that promotes reflections over ethics, morality, and political issues is fundamental for the growth of a future active citizen (Alfieri et al., 2013; Marta & Cristini, 2012; Marta, Marzana, Aresi, & Pozzi, 2016; Marta, Pozzi, & Marzana, 2010; Marzana, Marta, & Pozzi, 2012b). Interestingly, participants recalled a dimension of awareness, underlining the existence of a critical obedience in opposition to the well-known blind obedience. This result is an opportunity to stress the existence of constructive obedience, a fundamental feature of everyday life as it ensures social order (Darley, 1995).

Results from the current study showed a difference between Italian and U.S. American participants. Specifically, within the Italian sample, the SR of obedience evoked the *respect* to different types of *norms* and of *institutional authorities*. Here, obedience referred to norms that range on a continuum from oral (e.g., command by superiors) to written norms (e.g. constitutional laws, civil code). Again, a basic requisite for obedient behavior is the explication of the order, whether it is oral or written (Passini & Morselli, 2010). Moreover, written norms usually come from institutional authorities, legitimate organs that, in their efficient forms, are necessary and facilitate the administration of civil life (Passini & Morselli, 2009). U.S. American participants represented obedience differently also because of their association with themes such as *loyalty* and *personal respect*. The issue of loyalty connected to obedience was analyzed by studies on military authority relationship (Kelman & Hamilton, 1989; Pion-Berlin, Esparza, & Grisham, 2014). Analyzing obedience to superiors, Pion-Berlin et al. (2014) found that it was likely to happen when: “material interests were satisfied and where militaries identified with government, believed internal order missions to be appropriate, followed the law, and remained unified” (p. 246). Within the U.S. American sample, this theme was connected to the *ability* and *fear of punishment* components. Findings from the analysis of obedience behavior in military contexts revealed that military authority can also administer sanctions and punishment in order to ensure obedience (Kelman & Hamilton, 1989). Coherently, obedience was considered as an ability, something that a person needs to know how to enact. Moreover, Italians evaluated obedience neither positively nor negatively, while U.S. American participants conceived it as entirely positive. Given the recent debate on the military psychology (e.g. The British Psychological Society), it seems interesting to compare these results with those of a recent study on young Italian soldiers (manuscript submitted for publication). In fact, it appears that SR of (dis)obedience of Italian soldiers has similarities to the American civilians’ SR. Given these results, it seems interesting to study this topic and the connection between the two SR and deepen the debate.

#### Social Representation of Disobedience

The term “disobedience” evoked more themes and components than “obedience”. All the samples included the following themes in the social representation of disobedience: 1) Disobedience as *lack of norm respect*; 2) Disobedience as expression of *autonomy*; 3) *Different types of orders* convey disobedience; 4) *Awareness*, disobedience is always a chosen act; 5) *Punishment*, as a consequence of disobedience.

The issue of personal *autonomy* has been largely debated within the literature, from the eteronomy-autonomy dichotomy (Milgram, 1974) to the last reflections on civil disobedience (Moraro, 2014; Vecina, Marzana, & Paruzel-Czachura, 2015). According to these last insights, citizens are allowed to use a certain degree of force in opposition to coercion implemented by any other individual or institution. More precisely, it is reasonable to address moral autonomy (Leman & Duveen, 1999; Sonnentag & McDaniel, 2013) for positive disobedient actions, that is, the “inner” authority, composed by moral and ethical standards, opposed to the external authority. Accordingly, disobedience is evoked as a choice or result of an aware process and that requires more effort to be implemented (Sonnentag & McDaniel, 2013).

Disobedience evoked the theme of *punishment*, which, in this case, was seen as a consequence and not as a cause. In fact, disobedient people are often punished with retaliation by superiors (Berry, 2004), as well as with exclusion and stigmatization from their social groups (Milgram, 1974). Italians represented disobedience as *civil* and directed to an *institutional authority*. Civil disobedience is the most discussed type of disobedience since the reflections of the Greek myths such as Antigone, Lysistrata and Prometheus (Laudani, 2010). One of the defining features of disobedience is to be addressed by an institutional authority (Thoreau, 1849). Similarly, regarding what observed with the social representation of obedience, U.S. American participants considered disobedience as a lack of *personal respect* (loyalty) towards a person and as a *failure* (vs obedience as ability). Lastly, Italians evaluated disobedience according to the consequences of its implementation, while Americans considered it always negative.

### Conclusions

Social psychologists agree with the need to change the point of view on authority relationship, to *disobey* the traditional paradigm and methods used to inquire about obedience and disobedience (Morselli & Passini, 2012a; Pozzi et al., 2014). In this paper, the use of the SRT (Moscovici, 1961) provides a fundamental contribution in analyzing authority relationship from a societal level, examining it in a study on social representations of obedience and disobedience in U.S Americans and comparing it with previous research examining Italian participants. Scholars can give SR a more central role in studying this phenomenon, trying to integrate the study of isolated individuals with a more global look at relations. By doing this, it is possible to highlight how obedience and disobedience are not just behaviors, as clearly identified by multiple scholars across time (e.g., Milgram, 1963; Pozzi et al., 2014). Instead, obedience and disobedience are composed and can be considered as attitudes. Thus, this study allowed us to refer to the construct (dis)obedience, not only from a behavioral standpoint, but, instead, also evoking concepts related to affects and cognitions, which enabled us to defined it as an attitude, as inferred by the Multiple component model of attitude (ABCs of attitude by Eagly & Chaiken, 1993). According to this model, attitudes are the results of the influence of three components: affect (i.e. feelings and emotions elicited by the attitude object – in this case (dis)obedience), behavior (i. e. past events or experiences related to attitude object), and cognition (i.e. attributes, beliefs, thoughts related to the attitude object) (Aschieri et al., 2016). This approach allows the authors to place a new emphasis on the interpretation of the construct (dis)obedience as attitude, and, therefore, as an antecedent of behaviors. Based on this idea, it is possible to develop models able to explain human behaviors, in which (dis)obedience in one of his components is consider an attitude. Using Abric’s (2003) approach in the study of the SR of (dis)obedience, allowed the authors to identify what elements are central, as opposed to those that instead are peripheral, in the study of such a complex construct.

Summing up, if these two phenomena define the relationship with the authority and if the relationship with the authority is a central theme in one’s adequate social life, it is relevant to explore the meanings attributed to these social objects. This process will enable an understanding of the possible dynamics elicited by the relationship with the authority, whether this is an individual, as presented by the participants of this study, or an institution (Morselli & Passini, 2012b; Tamanza, Gozzoli, & Gennari, 2016).

The present paper also responds to the claim of Elcheroth, Doise, and Reicher (2011) calling for the study of social interactions through the use of “Social Representations”. Testing cross-cultural differences can also help researchers better understand interpersonal processes in different countries, especially when analyzing relationships individuals face with their authorities, with a more critical view of how obedience and disobedience are defined (i.e., commonly through Milgram’s 1963 lens).

Possible limitations of the current study include the use of a single instrument to evoke the social representations of obedience and disobedience. Even if this instrument is an effective, reliable, and time-saving tool to uncover social representations (Abric & Tafani, 2009; Fasanelli et al., 2005), interviewing the participants would have provided a deeper understanding of the content of the representations. In the future, it may also be fruitful to collect data from samples of other U.S. Americans, aiming to verify the validity of these results. New samples could confirm or disprove the absence of any levels of the *civil disobedience* component within disobedience social representation in the U.S.

As a possible development, authority relationship has been highlighted as a central theme in the study of civic participation (Mannarini, Roccato, Fedi, & Rovere, 2009; Morselli & Passini, 2012a), a type of social action that psychologists need to study from a societal point of view. Moreover, future studies could analyze social representations of authority relationship within groups of activist or members of social movements, to clarify both at theoretical and practical levels, their conceptions of authorities, policies, and social change (e.g., Fattori, Pozzi, et al., 2015; Aresi, Fattori, Pozzi, & Moore, 2016; Pozzi, Pistoni, & Alfieri, 2017).
